# Cross-Neutralisation of In Vitro Neurotoxicity of Asian and Australian Snake Neurotoxins and Venoms by Different Antivenoms

**DOI:** 10.3390/toxins8100302

**Published:** 2016-10-18

**Authors:** Anjana Silva, Wayne C. Hodgson, Geoffrey K. Isbister

**Affiliations:** 1 Monash Venom Group, Department of Pharmacology, Biomedicine Discovery Institute, Monash University, Clayton, VIC 3800, Australia; wayne.hodgson@monash.edu (W.C.H.); geoff.isbister@gmail.com (G.K.I.); 2 Faculty of Medicine and Allied Sciences, Rajarata University of Sri Lanka, Saliyapura 50008, Sri Lanka; 3 Clinical Toxicology Research Group, University of Newcastle, Newcastle, NSW 2298, Australia

**Keywords:** antivenom, cross-neutralisation, venom, neurotoxicity, snake

## Abstract

There is limited information on the cross-neutralisation of neurotoxic venoms with antivenoms. Cross-neutralisation of the in vitro neurotoxicity of four Asian and four Australian snake venoms, four post-synaptic neurotoxins (α-bungarotoxin, α-elapitoxin-Nk2a, α-elapitoxin-Ppr1 and α-scutoxin; 100 nM) and one pre-synaptic neurotoxin (taipoxin; 100 nM) was studied with five antivenoms: Thai cobra antivenom (TCAV), death adder antivenom (DAAV), Thai neuro polyvalent antivenom (TNPAV), Indian Polyvalent antivenom (IPAV) and Australian polyvalent antivenom (APAV). The chick biventer cervicis nerve-muscle preparation was used for this study. Antivenom was added to the organ bath 20 min prior to venom. Pre- and post-synaptic neurotoxicity of *Bungarus caeruleus* and *Bungarus fasciatus* venoms was neutralised by all antivenoms except TCAV, which did not neutralise pre-synaptic activity. Post-synaptic neurotoxicity of *Ophiophagus hannah* was neutralised by all antivenoms, and *Naja kaouthia* by all antivenoms except IPAV. Pre- and post-synaptic neurotoxicity of *Notechis scutatus* was neutralised by all antivenoms, except TCAV, which only partially neutralised pre-synaptic activity. Pre- and post-synaptic neurotoxicity of *Oxyuranus scutellatus* was neutralised by TNPAV and APAV, but TCAV and IPAV only neutralised post-synaptic neurotoxicity. Post-synaptic neurotoxicity of *Acanthophis antarcticus* was neutralised by all antivenoms except IPAV. *Pseudonaja textillis* post-synaptic neurotoxicity was only neutralised by APAV. The α-neurotoxins were neutralised by TNPAV and APAV, and taipoxin by all antivenoms except IPAV. Antivenoms raised against venoms with post-synaptic neurotoxic activity (TCAV) cross-neutralised the post-synaptic activity of multiple snake venoms. Antivenoms raised against pre- and post-synaptic neurotoxic venoms (TNPAV, IPAV, APAV) cross-neutralised both activities of Asian and Australian venoms. While acknowledging the limitations of adding antivenom prior to venom in an in vitro preparation, cross-neutralization of neurotoxicity means that antivenoms from one region may be effective in other regions which do not have effective antivenoms. TCAV only neutralized post-synaptic neurotoxicity and is potentially useful in distinguishing pre-synaptic and post-synaptic effects in the chick biventer cervicis preparation.

## 1. Introduction

Snakebites impose a considerable health and socioeconomic burden on many nations in southern and south-eastern Asian. This is due to the large number of cases of envenomings that cause acute, life-threatening, and debilitating long-term consequences [1,2,3,4]. Neuromuscular paralysis due to snake envenoming is a common important complication because of the rapid development of bulbar and respiratory muscle paralysis that requires early and often prolonged intervention with intubation and mechanical ventilation [5]. Envenoming by snakes such as kraits (genus *Bungarus*), some species of cobras (genera *Naja* and *Ophiophagus*), taipans (genus *Oxyuranus*), death adders (genus *Acanthophis*), tiger snakes (genus *Notechis*) and coral snakes (genus *Micrurus*) commonly leads to life threatening neuromuscular paralysis [5].

In vitro and in vivo characterisation of neurotoxic venoms and isolated neurotoxins has been useful for understanding the pathophysiology of neuromuscular dysfunction in snake envenoming [6]. Neurotoxins from medically important snakes act either pre-synaptically or post-synaptically at the neuromuscular junction. Snake venom pre-synaptic neurotoxins (i.e., β-neurotoxins) are usually phospholipase A_2_ (PLA_2_) toxins. They enter the motor nerve terminal and lead to a depletion of synaptic vesicles by facilitating exocytosis and inhibiting synaptic vesicle recycling. This is followed by the rapid degeneration of the motor nerve terminal which is not reversible with treatment [7,8]. Snake venom post-synaptic toxins (i.e., α-neurotoxins) possess a three finger toxin structure and have either 61–62 amino acids with four disulphide bonds (i.e., short-chain toxins) or 64–71 amino acids with five disulphide bonds (i.e., long-chain toxins). The primary amino acid sequence of these toxins shows significant homology across diverse species [9,10]. Post-synaptic toxins bind to the two agonist binding sites of the nicotinic acetylcholine receptors (nAChR) at the motor end plate with high affinity, and prevent the opening of the associated ion channel, blocking neurotransmission. Venoms of many medically important neurotoxic snakes such as kraits and Australasian elapids have both pre- and post-synaptic toxins in their venoms [6,7,8,9,11]. Although the clinical importance of the pre-synaptic toxins in causing neuromuscular paralysis in snake envenomed humans is well recognised, the importance of post-synaptic toxins in human envenoming remains unclear [8,12].

Antivenoms have been used to treat snake envenomings for more than a century, and include monovalent and polyvalent antivenoms developed to treat neurotoxic envenoming. Irrespective of the wide diversity of snakes, snake venoms share many common toxin groups, including neurotoxic PLA_2_ and three finger toxins [7,9]. Despite the structural and functional variations of the toxins within these toxin groups, similarities in their immunogenicity lead to cross-neutralisation by antivenoms raised against different snakes. Cross-neutralisation has been previously reported for some venom and antivenom combinations [13,14,15,16,17,18,19]. In most previous studies examining neurotoxic elapid venoms and antivenoms, cross-neutralisation has been measured by means of the prevention of rodent lethality (ED_50_) [15,16,20]. However, death in these ‘envenomed’ animals is almost certainly a summative effect of the many toxins. Therefore, lethality tests are not always useful in understanding the ability of the antivenoms to specifically cross-neutralise the most clinically important effect—neurotoxicity [21]. Furthermore, in investigating the cross-neutralisation of neurotoxicity it is essential to understand the type of neurotoxicity (i.e., pre-synaptic or post-synaptic) that is being cross-neutralised by the antivenom, given that many snake venoms contain both types of neurotoxins. It is therefore necessary to further investigate this phenomenon using venoms from a range of neurotoxic snakes, neurotoxins and antivenoms.

The aim of this study was to test the ability of antivenoms raised against neurotoxic venoms to cross-neutralise individual neurotoxins and whole venoms of other neurotoxic snakes in vitro.

## 2. Results

The neurotoxicity of the venoms ranged from a t_90_ (i.e., time to 90% inhibition of twitch height) of 15.0 ± 2.1 min (*Pseudonaja textillis*), which is highly potent, to a t_90_ of 128.0 ± 17.2 min (*O. scutellatus*; Supplementary Table S1).

### 2.1. Cross-Neutralisation of Asian Neurotoxic Snake Venoms by Different Antivenoms

#### 2.1.1. *Bungarus caeruleus*

**Figure 1:**
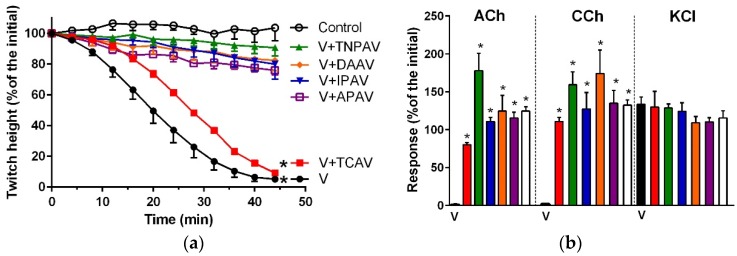
Cross-neutralisation of the neurotoxicity of *Bungarus caeruleus* venom (5 µg/mL, V, black) by Thai cobra antivenom (TCAV; red), death adder antivenom (DAAV; orange), Thai neuro polyvalent antivenom (TNPAV; green), Indian polyvalent antivenom (IPAV; blue), and Australian polyvalent antivenom (APAV; purple) in the chick biventer nerve-muscle preparation compared to control (white): (**a**) Prevention of the inhibition of indirect twitches caused by venom (V). Note: all antivenoms except TCAV prevented neurotoxicity (* significantly different from control at 44 min, *p* < 0.05, one-way ANOVA followed by Bonferroni’s post-hoc test); (**b**) The effect of venom on response to exogenous agonists (ACh, CCh and KCl) in the absence of antivenom and the presence of antivenoms compared to control. Note: all antivenoms prevented the abolition of agonist responses by the venom (* significantly different from the venom, *p* < 0.05, one-way ANOVA followed by Bonferroni’s post-hoc test).

**Figure 2:**
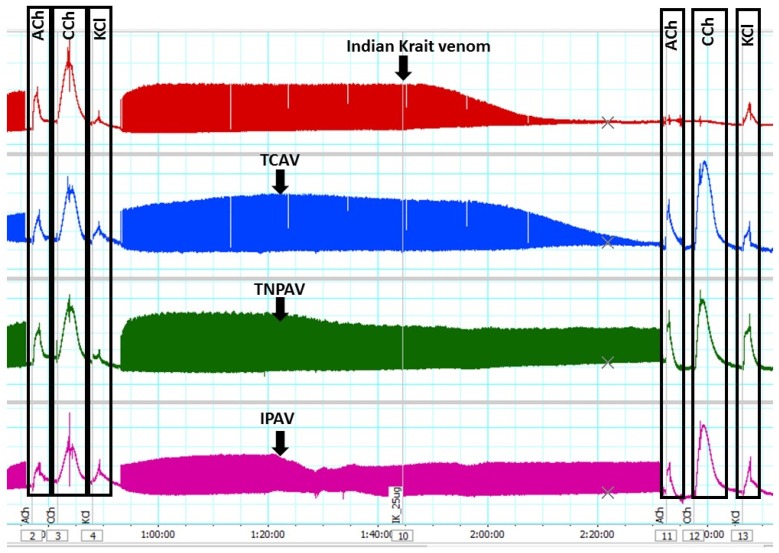
Traces showing indirect twitch and agonist contractile responses of the chick-biventer nerve-muscle preparation for *B. caeruleus* venom (5 µg/mL) in the absence and the presence of Thai cobra antivenom (TCAV), Thai neuro polyvalent antivenom (TNPAV) and Indian polyvalent antivenom (IPAV). Note: the venom (red trace) inhibits indirect twitches and abolishes ACh and CCh responses indicating post-synaptic neurotoxicity. In the presence of TCAV (blue trace), the venom inhibits indirect twitches but does not abolish ACh and CCh responses indicating that the pre-synaptic neurotoxicity is not neutralised. In the presence of TNPAV (green trace) and IPAV (purple trace), venom fails to inhibit indirect twitches and fails to abolish ACh and CCh responses indicating neutralisation of both pre- and post-synaptic neurotoxicity of the venom.

*B. caeruleus* venom (5 µg/mL) inhibited indirect twitches (Figure 1a and Figure 2) and abolished the response of the chick biventer preparation to exogenous nicotinic receptor agonists—acetylcholine (ACh) and carbachol (CCh)—indicating the venom had post-synaptic neurotoxicity (Figure 1b and Figure 2). Thai cobra antivenom (TCAV) did not prevent the inhibition of indirect twitches but prevented the abolition of the response of the chick biventer preparation to exogenous nicotinic agonists, indicating the neutralisation of post-synaptic effects, but not the pre-synaptic neurotoxicity of the venom. Indian polyvalent antivenom (IPAV), Thai neuro polyvalent antivenom (TNPAV), death adder antivenom (DAAV), and Australian polyvalent antivenom (APAV) all prevented the inhibition of indirect twitches, and the abolition of the response of the chick biventer preparation to exogenous nicotinic receptor agonists, indicating the neutralisation of both pre- and post-synaptic effects of the venom (Figure 1b and Figure 2).

#### 2.1.2. *Bungarus fasciatus*

**Figure 3:**
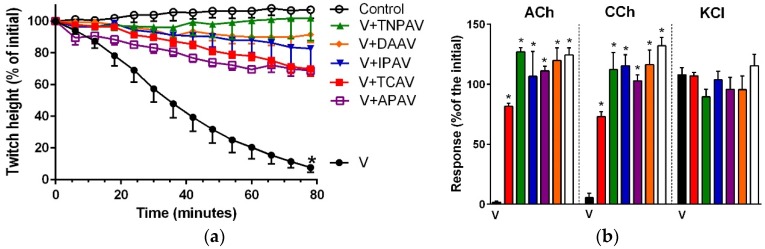
Cross-neutralisation of the neurotoxicity of *Bungarus fasciatus* venom (7.5 µg/mL, V, black) by Thai cobra antivenom (TCAV; red), death adder antivenom (DAAV; orange), Thai neuro polyvalent antivenom (TNPAV; green), Indian polyvalent antivenom (IPAV; blue), and Australian polyvalent antivenom (APAV; purple) in the chick biventer nerve-muscle preparation compared to control (white): (**a**) Prevention of the inhibition of indirect twitches caused by venom. Note: all antivenoms prevented neurotoxicity (twitch height as a percentage of the initial at 78 min not different from the control, one-way ANOVA followed by Bonferroni’s post-hoc test); (**b**) The effect of venom on response to exogenous agonists (ACh, CCh and KCl) in the absence of antivenom and the presence of antivenoms compared to control. Note: all antivenoms prevented the abolition of agonist responses by the venom (* significantly different from the venom, *p* < 0.05, one-way ANOVA followed by Bonferroni’s post-hoc test).

*B. fasciatus* venom (7.5 µg/mL) inhibited indirect twitches (Figure 3a) and abolished the response of the chick biventer preparation to exogenous nicotinic receptor agonists, indicating post-synaptic neurotoxic activity of the venom (Figure 3b). All antivenoms prevented the inhibition of indirect twitches (Figure 3a) and the abolition of the response of chick biventer preparation towards exogenous agonists (Figure 3b).

#### 2.1.3. *Ophiophagus hannah*

**Figure 4:**
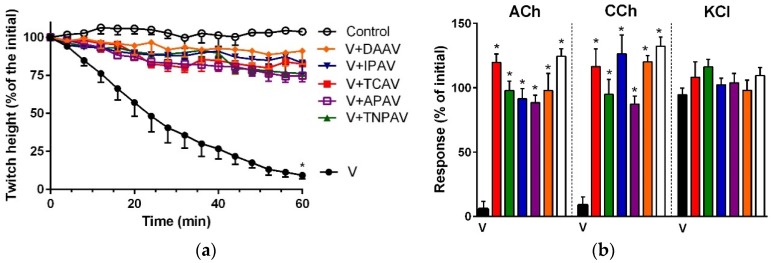
Cross-neutralisation of the neurotoxicity of *Ophiophagus hannah* venom (5 µg/mL, V, black) by, Thai cobra antivenom (TCAV; red), death adder antivenom (DAAV; orange), Thai neuro polyvalent antivenom (TNPAV; green), Indian polyvalent antivenom (IPVA), and Australian polyvalent antivenom (APAV) in chick biventer nerve-muscle preparation compared to control (white): (**a**) Prevention of the inhibition of indirect twitches caused by venom (V). Note: all antivenoms prevented neurotoxicity (twitch height as a percentage of the initial at 60 min not different from the control, one-way ANOVA followed by Bonferroni’s post-hoc test); (**b**) The effect of venom on the response to exogenous agonists (ACh, CCh and KCl) in the absence of antivenom and the presence of antivenoms compared to control. Note: all antivenoms prevented the abolition of agonist responses by the venom (* significantly different from venom, *p* < 0.05, one-way ANOVA followed by Bonferroni’s post-hoc test).

*O. hannah* venom (5 µg/mL) inhibited indirect twitches (Figure 4a) and abolished the response of the chick biventer preparation to exogenous nicotinic receptor agonists, indicating post-synaptic neurotoxic activity of the venom (Figure 4b). All tested antivenoms prevented the inhibition of indirect twitches (Figure 4a) and the abolition of the response of the chick biventer preparation to exogenous agonists (Figure 4b).

#### 2.1.4. *Naja kaouthia*

**Figure 5:**
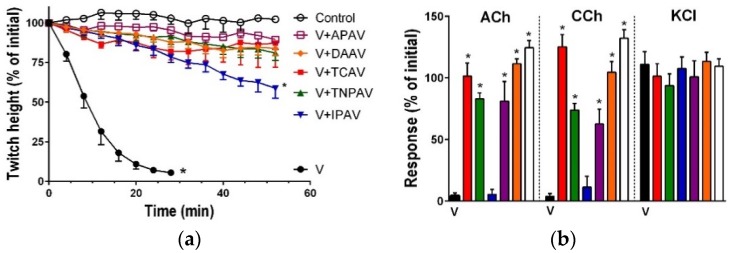
Cross-neutralisation of the neurotoxicity of *Naja kaouthia* venom (5 µg/mL, V, black) by Thai cobra antivenom (TCAV; red), death adder antivenom (DAAV; orange), Thai neuro polyvalent antivenom (TNPAV; green), Indian polyvalent antivenom (IPVA), and Australian polyvalent antivenom (APAV) in chick biventer nerve-muscle preparation compared to control (white): (**a**) Prevention of the inhibition of indirect twitches caused by venom (V). Note: all antivenoms except IPAV prevented neurotoxicity (* significantly lower from the control at 44 min, *p* < 0.05, one-way ANOVA followed by Bonferroni’s post-hoc test); (**b**) The effect of venom on the response to exogenous agonists (ACh, CCh and KCl) in the absence of antivenom and presence of antivenoms compared to control. Note: all antivenoms except IPAV prevented the abolition of agonist responses by the venom (* significantly different from the venom, *p* < 0.05, one-way ANOVA followed by Bonferroni’s post-hoc test).

*N. kaouthia* venom (5 µg/mL) inhibited indirect twitches (Figure 5a) and abolished the response of the chick biventer preparation to exogenous nicotinic receptor agonists, indicating post-synaptic neurotoxic activity of the venom (Figure 5b). All tested antivenoms, except IPAV, prevented the inhibition of indirect twitches (Figure 5a) and abolition of the response of the chick biventer preparation towards exogenous agonists (Figure 5b). IPAV partially prevented the twitch inhibition, with the twitch force dropping by 50% over 60 min in the presence of antivenom (Figure 5a). However, the abolition of the response of chick biventer preparation towards exogenous agonists by the venom was not prevented by IPAV (Figure 5b).

### 2.2. Cross-Neutralisation of the Neurotoxicity of Australasian Elapid Venoms by Different Antivenoms

#### 2.2.1. *Notechis scutatus*

**Figure 6:**
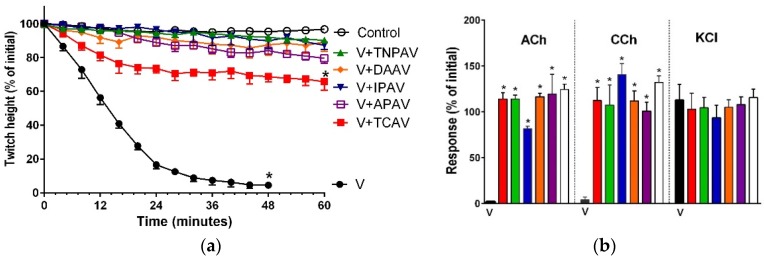
Cross-neutralisation of the neurotoxicity of *Notechis scutatus* venom (5 µg/mL, V, black) by Thai cobra antivenom (TCAV; red), Thai neuro polyvalent antivenom (TNPAV; green), Indian polyvalent antiveom (IPAV; blue), death adder antivenom (DAAV; orange), and Australian polyvalent antivenom (APAV; purple) in chick biventer nerve-muscle preparation compared to control: (**a**) Prevention of the inhibition of indirect twitches caused by venom (V). Note: all antivenoms except TCAV prevented neurotoxicity (* significantly lower from the control at 48 min, *p* < 0.05, one-way ANOVA followed by Bonferroni’s post-hoc test); (**b**) The effect of *N. scutatus* venom on the response to exogenous agonists (ACh, CCh and KCl) in the absence and the presence of antivenoms compared to controls. Note: all antivenoms prevented the abolition of agonist responses by the venom (* significantly different from the venom, *p* < 0.05, one-way ANOVA followed by Bonferroni’s post-hoc test).

*N. scutatus* venom (5 µg/mL) inhibited indirect twitches (Figure 6a) and abolished the response of the chick biventer preparation to exogenous nicotinic receptor agonists, indicating post-synaptic neurotoxicity of the venom (Figure 6b). TNPV, IPAV and APAV prevented venom-mediated inhibition of indirect twitches (Figure 6a) and abolition of the agonist responses in the chick biventer preparation (Figure 6b). TCAV only partially prevented the indirect twitch inhibition with the twitch inhibition being <45% after 48min (Figure 6a) but completely prevented abolishment of the response of chick biventer preparation to exogenous agonists (Figure 6b). This suggests that TCAV only blocks the post-synaptic effect of *N. scutatus* venom.

#### 2.2.2. *Oxyuranus scutellatus*

**Figure 7:**
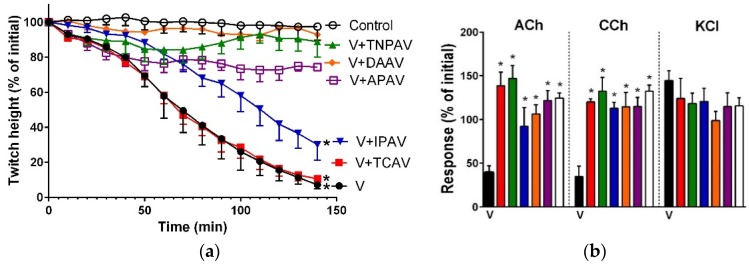
Cross-neutralisation of the neurotoxicity of *Oxyuranus scutellatus* venom (5 µg/mL, V, black) by Thai cobra antivenom (TCAV; red), Thai neuro polyvalent antivenom (TNPAV; green), Indian polyvalent antivenom (IPAV; blue), death adder antivenom (DAAV; orange), and Australian polyvalent antivenom (APAV; purple) in the chick biventer nerve-muscle preparation compared to control: (**a**) Prevention of the inhibition of indirect twitches caused by venom (V). Note: TCAV and IPAV failed to prevent neurotoxicity (twitch height as a percentage of the initial at 140 min is different from the control, one-way ANOVA followed by Bonferroni’s post-hoc test); (**b**) The effect of venom on the response to exogenous agonists (ACh, CCh and KCl) in the absence and the presence of antivenoms compared to controls. Note: all antivenoms prevented the inhibition of agonist responses by the venom (* significantly different from the venom, *p* < 0.05, one-way ANOVA followed by Bonferroni’s post-hoc test).

*O. scutellatus* venom (5 µg/mL) inhibited indirect twitches (Figure 7a), but only partially inhibited the response of the chick biventer preparation to exogenous nicotinic receptor agonists (Figure 7b) suggesting that the neurotoxicity of *O. scutellatus* venom is mainly due to pre-synaptic effects [1]. TNPAV and APAV prevented venom-mediated inhibition of indirect twitches (Figure 7a), and prevented the abolition of the exogenous nicotinic agonist responses of the chick biventer preparation (Figure 7b). TCAV and IPAV were unable to prevent the inhibition of indirect twitches, although IPAV appeared to partially prevent inhibition of indirect twitches by 70% (Figure 7a). However, both TCAV and IPAV prevented abolishment of the response of chick biventer preparation to exogenous nicotinic agonists (Figure 7b), indicating that the two antivenoms appeared to be able to neutralize post-synaptic but not pre-synaptic effects of the venom.

#### 2.2.3. *Acanthophis antarcticus*

**Figure 8:**
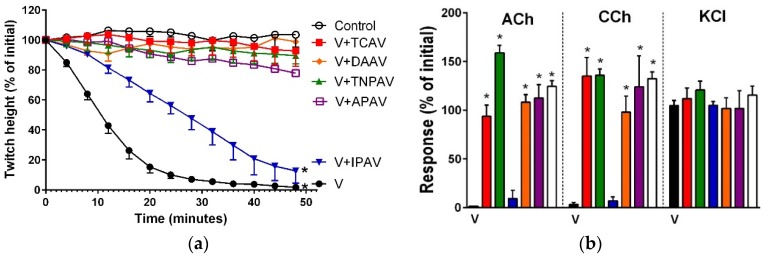
Cross-neutralisation of the neurotoxicity of *Acanthophis antarcticus* venom (5 µg/mL, V, black) by Thai cobra antivenom (TCAV; red), Thai neuro polyvalent antivenom (TNPAV; green), Indian polyvalent antivenom (IPAV; blue), death adder antivenom (DAAV; orange), Australian polyvalent antivenom (APAV; purple) in chick biventer nerve-muscle preparation compared to control: (**a**) Prevention of the inhibition of indirect twitches caused by venom (V). Note: TCAV and IPAV failed to prevent neurotoxicity (twitch height as a percentage of the initial at 48 min is different from the control, one-way ANOVA followed by Bonferroni’s post-hoc test); (**b**) The effect of venom on the response to exogenous agonists (ACh, CCh and KCl) in the absence and the presence of antivenoms compared to controls. Note: all antivenoms prevented the inhibition of agonist responses by the venom (* significantly different from the venom, *p* < 0.05, one-way ANOVA followed by Bonferroni’s post-hoc test).

*A. antarcticus* venom (5 µg/mL) inhibited indirect twitches (Figure 8a), and abolished the response of the chick biventer preparation to exogenous nicotinic receptor agonists (Figure 8b) indicating post-synaptic neurotoxicity of the venom. All antivenoms except IPAV prevented the venom-mediated abolition of indirect twitches (Figure 8a) and the response of the chick biventer preparation towards exogenous nicotinic receptor agonists (Figure 8b). IPAV failed to prevent the inhibition of indirect twitches, with 85% of the twitch height still inhibited at 48 min (Figure 8a), and also did not prevent abolishment of the response of the chick biventer preparation to exogenous agonists (Figure 8b). This suggests that IPAV was unable to neutralise the post-synaptic neurotoxic effects of *A. antarcticus* venom.

#### 2.2.4. *Pseudonaja textillis*

**Figure 9:**
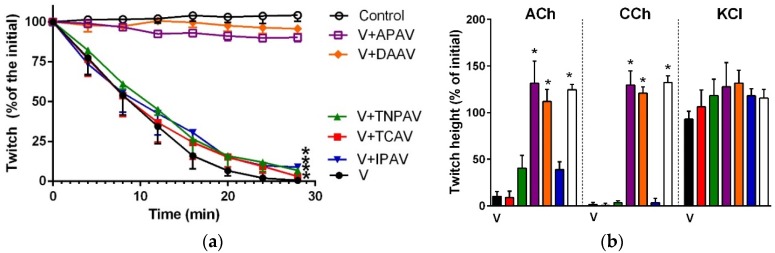
Cross-neutralisation of the neurotoxicity of *Pseudonaja textillis* venom (5 µg/mL, V, black) by Thai cobra antivenom (TCAV; red), Thai neuro polyvalent antivenom (TNPAV; green), Indian polyvalent antivenom (IPAV; blue), death adder antivenom (DAAV; orange), and Australian polyvalent antivenom (APAV; purple) in the chick biventer nerve-muscle preparation compared to control (white): (**a**) Prevention of the inhibition of indirect twitches caused by venom (V). Note: only APAV prevented neurotoxicity (* twitch height as a percentage of the initial at 28 min significantly different from the control, one-way ANOVA followed by Bonferroni’s post-hoc test); (**b**) The effect of *P. textillis* venom on the response to exogenous nicotinic receptor agonists (ACh, CCh and KCl) in the absence and the presence of antivenoms. Note: only APAV prevented the abolition of ACh and CCh responses by the venom (* significantly different from the venom, *p* < 0.05, one-way ANOVA followed by Bonferroni’s post-hoc test).

*P. textillis* venom (5 µg/mL) abolished indirect twitches (Figure 9a) and abolished the response of the chick biventer preparation towards exogenous nicotinic receptor agonists (Figure 9b) indicating post-synaptic neurotoxicity of the venom. APAV prevented the venom-mediated inhibition of indirect twitches (Figure 9a) and abolishment of the response of nerve-muscle preparation to exogenous nicotinic receptor agonists (Figure 9b). TCAV, TNPAV and IPAV all failed to prevent the inhibition of the venom-mediated indirect twitches (Figure 9a). TCAV and IPAV did not prevent the abolishment of the response of the chick biventer preparation to exogenous agonists, (Figure 9b) and TNPAV partially prevented the abolishment of the response of the chick biventer preparation to exogenous agonists (Figure 9b).

**Figure 10:**
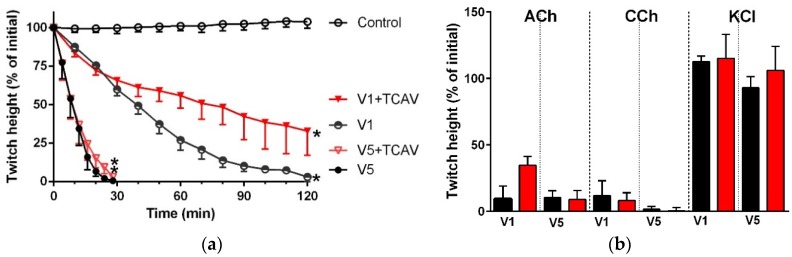
Cross-neutralisation of the neurotoxicity of 1 µg/mL vs. 5 µg/mL *P. textillis* venom by Thai cobra antivenom (TCAV; red): (**a**) Inhibition of indirect twitches caused by 1 µg/mL (V1) vs. 5 µg/mL (V5) *P. textillis* venom in the presence of TCAV. Note: TCAV does not prevent the twitch abolition caused by 1 µg/mL *P. textillis* venom (* significantly lower from the control at 120 min, *p* < 0.05, one-way ANOVA followed by Bonferroni’s post-hoc test); (**b**) The effect of 1 µg/mL (V1) vs. 5 µg/mL (V5) *P. textillis* venom on the response to exogenous nicotinic agonists (ACh, CCh and KCl) in the absence and presence of TCAV. Note: TCAV failed to prevent the abolition of agonist responses caused by 1 µg/mL venom (no significant difference from the venom, one-way ANOVA followed by Bonferroni’s post-hoc test).

At a lower dose of *P. textillis* venom (1 µg/mL), the inhibition of indirect twitches was not prevented by TCAV (i.e., twitch height not different from the venom group at any time point; *p* < 0.05, unpaired t test; Figure 10a). Furthermore, the venom (1 µg/mL) mediated abolition of the response of the chick biventer preparation towards exogenous nicotinic receptor agonists was not prevented by TCAV (*p* < 0.05, one-way ANOVA followed by Bonferroni’s post hoc test; Figure 10b).

This suggests that there was little cross-neutralisation between *P. textillis* neurotoxicity and Asian antivenoms.

### 2.3. Cross-Neutralisation of Snake Venom Neurotoxins by Antivenoms

#### 2.3.1. α-Neurotoxins: α-Bungarotoxin, α-Elapitoxin-Nk2a, α-Elapitoxin-Ppr1 and α-Scutoxin

**Figure 11:**
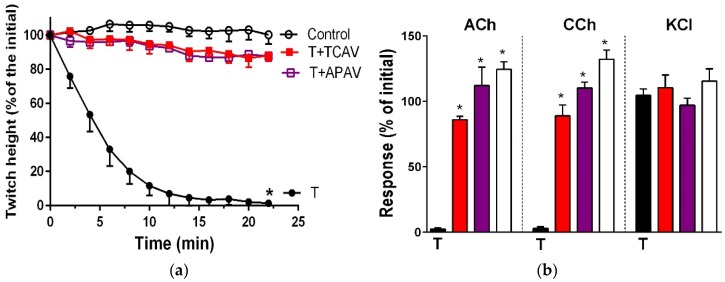

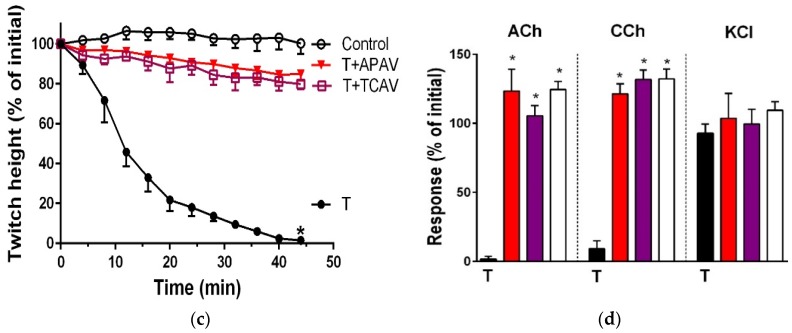
Cross-neutralisation of the neurotoxicity of the long-chain α-neurotoxins (α-bungarotoxin and α-elapitoxin-Nk2a; black) by Thai cobra antivenom (TCAV; red) and Australian polyvalent antivenom (APAV; purple) in the chick biventer nerve-muscle preparation compared to control (white): (**a**) Prevention of the inhibition of indirect twitches caused by 100 nM α-bungarotoxin (T). Note: APAV and TCAV effectively prevented neurotoxicity (no difference from the control at 22 min, one-way ANOVA followed by Bonferroni’s post-hoc test); (**b**) The effect of α-bungarotoxin on response to exogenous nicotinic receptor agonists (ACh, CCh and KCl) in the absence and the presence of antivenoms. Note: APAV and TCAV prevented the abolition of agonist responses caused by the toxin (* significantly different from the venom, *p* < 0.05, one-way ANOVA followed by Bonferroni’s post-hoc test); (**c**) Prevention of the inhibition of indirect twitches caused by 100 nM α-elapitoxin-Nk2a (T). Note: APAV and TCAV prevented neurotoxicity (twitch height as a percentage of the initial at 44 min not different from the control, one-way ANOVA followed by Bonferroni’s post-hoc test); (**d**) The effect of 100 nM α-elapitoxin-Nk2a on response to exogenous agonists (ACh, CCh and KCl) in the presence of antivenoms. Note: APAV and TCAV prevented the abolition of agonist responses by the venom (* significantly different from the toxin, *p* < 0.05, one-way ANOVA followed by Bonferroni’s post-hoc test).

**Figure 12:**
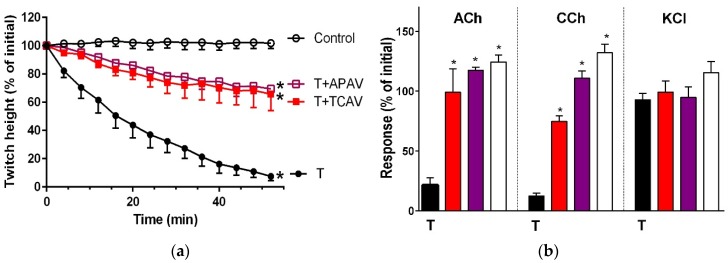

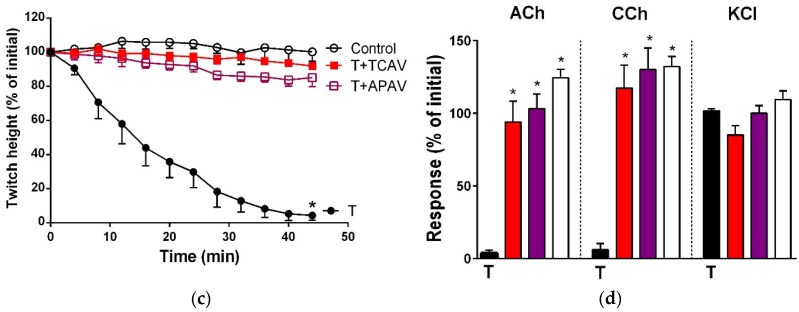
Cross-neutralisation of the neurotoxicity of the short-chain α-neurotoxins (α-scutoxin and α-elapitoxin-Ppr-1; T, black) by Thai cobra antivenom (TCAV; red) and Australian polyvalent antivenom (APAV; purple) in chick biventer nerve-muscle preparation compared to control (white); (**a**) Prevention of the inhibition of indirect twitches caused by α-scutoxin (T). Note: APAV and TCAV partially prevented neurotoxicity (* significantly lower from the control and the toxin at 54 min, *p* < 0.05, one-way ANOVA followed by Bonferroni’s post-hoc test); (**b**) The effect of 100 nM α-scutoxin on the response to exogenous nicotinic receptor agonists (ACh, CCh and KCl) in the absence and the presence of antivenoms compared to control. Note: APAV and TCAV prevented the abolition of agonist responses caused by the toxin (* significantly different from the venom, *p* < 0.05, one-way ANOVA followed by Bonferroni’s post-hoc test); (**c**) Prevention of the inhibition of indirect twitches caused by 100 nM α-elapitoxin-Ppr-1 (T). Note: APAV and TCAV prevented neurotoxicity (twitch height as a percentage of the initial at 44 min not different from the control, one-way ANOVA followed by Bonferroni’s post-hoc test); (**d**) The effect of 100 nM α-elapitoxin-Ppr1 on the response to exogenous nicotinic receptor agonists (ACh, CCh and KCl) in the absence and the presence of antivenoms compared to control. Note: APAV and TCAV prevented the abolition of agonist responses by the venom (* significantly different from the toxin, *p* < 0.05, one-way ANOVA followed by Bonferroni’s post-hoc test).

Indirect twitches and the response to exogenous nicotinic receptor agonists of the chick biventer nerve-muscle preparation were abolished by the two long-chain α-neurotoxins, α-bungarotoxin (Figure 11: panels a and b) and α-elapitoxin-Nk2a (Figure 11: panels c and d), and the two short-chain α-neurotoxins, α-elapitoxin-Ppr1 (Figure 12: panels a and b) and α-scutoxin (Figure 12: panels c and d) at 100 nM. APAV and TCAV both fully neutralised the effects of all the toxins except α-scutoxin (Figure 11, panels a and c; Figure 12, panel c), which was only partially neutralised, in terms of the prevention of twitch inhibition by the two antivenoms (Figure 12, panel a).

#### 2.3.2. β-Neurotoxin: Taipoxin

**Figure 13:**
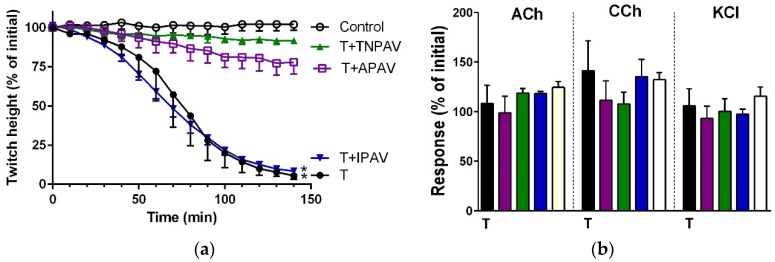
Cross-neutralisation of the neurotoxicity of the pre-synaptic neurotoxin from *Oxyuranus scutellatus*, taipoxin (100 nM, T, black), by Thai neuro polyvalent antivenom (TNPAV, green), Indian polyvalent antivenom (IPAV, blue) and Australian polyvalent antivenom (APAV, orange) in the chick biventer nerve-muscle preparation: (**a**) Prevention of the inhibition of indirect twitches caused by 100 nM taipoxin (T). Note: All antivenoms except IPAV effectively prevented the neurotoxicity (* significant difference from the control at 140 min, One-way ANOVA followed by Bonferroni’s post-hoc test); (**b**) The effect of taipoxin on the response to exogenous agonists (ACh, CCh and KCl) in the absence and the presence of antivenoms compared to control. Note: neither the toxin alone nor the toxin in the presence of antivenoms was able to abolish the agonist responses (no significant difference among groups, one-way ANOVA followed by Bonferroni’s post-hoc test).

Taipoxin (100 nM) abolished indirect twitches of the chick biventer nerve-muscle preparation (Figure 13a), but did not abolish the response of the preparation to exogenous nicotinic receptor agonists (Figure 13b). IPAV failed to prevent the toxin-mediated twitch inhibition, but both APAV and TNPAV were able to neutralise this effect (Figure 13a).

## 3. Discussion

This study has shown cross-neutralisation of the in vitro neurotoxicity of eight elapid venoms from Asia and Australia, four α-neurotoxins and one β-neurotoxin, using five Asian and Australian antivenoms. This demonstrates remarkable cross-neutralisation of snake venom neurotoxicity, both at a pre-synaptic and post-synaptic level, by a range of antivenoms raised against geographically distinct elapids. With the exception of *P. textillis* venom, the post-synaptic neurotoxicity of all tested venoms and toxins was neutralised by TCAV. Both pre- and post-synaptic neurotoxicity of all the tested venoms and toxins was neutralised by TNPAV, APAV and DAAV. IPAV was able to neutralise some of the pre- and post-synaptic neurotoxic effects of the tested venoms.

Although the effectiveness of antivenoms in treating established neurotoxic envenoming in humans has been questioned for many snakes, these antivenoms are clearly efficacious in preventing in vitro neurotoxicity and have been shown to bind with circulating venom [22,23,24,25]. Snake antivenoms contain polyclonal antibodies—Fab or F(ab’)_2_ fragments—purified from serum of animals immunised against snake venoms [26]. Depending on the antigenicity of the different constituent toxins and their relative abundance in the venom, or the venoms used in the immunogen mixture, the antibodies against each toxin type can be found within the antivenoms [27]. The presence of common antigenic regions leads to cross-neutralisation of the snake venom toxins by antibodies raised against different toxins [28]. This occurs most commonly with toxins of the same type (e.g., phospholipases, snake venom metalloproteinases). α-Neurotoxins display a high degree of homology at some key regions in their primary structure, especially related to the residues essential for their post-synaptic activity [29,30]. Of the α-neurotoxins, both short-chain (type I) and long-chain (type II) toxins, which are present in Australasian and Asian elapid venoms, share some key structural characteristics. However, Type III toxins, which are structurally distinct from the former two groups, are only present in the venom of brown snakes (*Pseudonaja* spp.) [9,29]. Investigation of the structural determinants of the common antigenic regions in α-neurotoxins is currently not available. However, it is not unreasonable to assume that the remarkable cross-neutralisation of post-synaptic neurotoxicity seen in this study is due to the sharing of common antigenic regions across a range of α-neurotoxins. The inability of all tested antivenoms, except APAV and DAAV, (which contain antibodies against *P. textillis* venom) [31], to neutralise the post-synaptic neurotoxicity of *P. textillis* venom is most likely due to the inability of Asian antivenoms to cross-neutralise the type III α-neurotoxins only present in *P. textillis* venom. This is further supported by the observation that, when the concentration of *P. textillis* venom was lowered by a factor of five, while maintaining the same concentration of TCAV, the antivenom was still unable to prevent post-synaptic neurotoxicity.

PLA_2_ neurotoxins in their monomeric form are generally 13 to 15 kDa molecules and display high homology in their primary sequences [7]. Presynaptic neurotoxic proteins with PLA_2_ activity, together with other proteins with or without PLA_2_ activity, are most commonly multi-subunit toxins. Notexin is a monomer from *N. scutatus* venom; taipoxin is a trimer from *O. scutellatus* venom; β-bungarotoxin is a heterodimer from Chinese banded krait (*Bungarus multicinctus*) venom, and textilotoxin is a pentamer from eastern *P. textillis* venom; all are examples of biochemically well characterized pre-synaptic toxins [7,32]. Irrespective of the structural differences in toxins, the effective cross-neutralisation of the pre-synaptic neurotoxicity of all venoms and toxins by TNPAV, APAV, DAAV and IPAV is most likely due to the sharing of common antigenic regions among different pre-synaptic toxins, similar to what is speculated for the α-neurotoxins.

IPAV was able to effectively neutralise both pre- and post-synaptic neurotoxicity of *N. scutatus* venom, *B. fasciatus* and the post-synaptic activity of *O. hannah* venom. Previously, it has been reported that 8.3 µL of IPAV effectively neutralised the in vivo neuromuscular blocking effects (in anaesthetised rats) of 1 µg *N. kaouthia* venom [15]. However in the current study, 2.8 µL of 5 times concentrated IPAV (equivalent to 14 µL of the standard solution) failed to effectively neutralise the post-synaptic neurotoxicity of 1 µg *N. kaouthia* venom. Further, IPAV failed to neutralise the neurotoxicity of *A. antarcticus* and *O. scutellatus* venom as well as taipoxin. This is most likely because the tested antivenom amounts were small in proportion to the venoms being used and therefore insufficient to fully cross-neutralise neurotoxins. This may have been due to the poor efficacy of the antivenom batch used. IPAV has been reported to have poor efficacy and there is considerable inter-batch variation of the actual protein content in each vial [21].

Of the in vitro nerve-muscle preparations, the chick-biventer nerve-muscle preparation has advantages compared to other preparations due to the uncomplicated dissection and, most importantly, the ability of the muscle to contract in response to exogenous nicotinic receptor agonists. This enables the preparation to be used to differentiate between pre- and post-synaptic neurotoxicity [6,33]. The chick biventer preparation is also beneficial for testing the efficacy of antivenoms against neurotoxins in venoms [18,33]. To elicit contractile responses to ACh and CCh, which is required to be able to demonstrate a pre-synaptic site of action, nAChR at the motor end plate must be functioning. So, when both pre- and post-synaptic toxins are present in a venom, the post-synaptic neurotoxins block the nAChR, making it impossible to detect pre-synaptic neurotoxicity. Further, post-synaptic toxins act faster than pre-synaptic toxins, making the pre-synaptic neurotoxicity even harder to identify [6]. Interestingly, in the current study, TCAV effectively neutralised the post-synaptic neurotoxicity of all the venoms but failed to neutralise the pre-synaptic effects of *B. caeruleus*, *B. fasciatus*, *O. scutellatus* and *N. scutatus* venoms. These venoms all cause paralysis in humans and are known to contain pre-synaptic toxins [11,18,34,35,36].

The recently published comparative venom proteome of *N. kaouthia* found that 78% of the venom consists of three finger toxins, including long-chain α-neurotoxins, that make up 33% of the venom. However, no toxins similar to pre-synaptic neurotoxins, such as basic PLA_2_s, were identified [37]. Therefore, it could be assumed that TCAV possesses high relative abundance of antibodies against post-synaptic neurotoxins, and may contain very few or no antibodies against toxins with pre-synaptic neurotoxicity. This means that by pre-incubating venoms with TCAV, the antibodies raised against α-neurotoxins in the antivenom would bind any α-neurotoxins, allowing the pre-synaptic neurotoxicity of the venom to be studied in the chick-biventer preparation.

Pre-synaptic neurotoxins similar to β-bungarotoxin are present in *B. fasciatus* venom [35]. However, such activity was not prominent in this study, even with the presence of TCAV. This is probably because of a combination of the low relative abundance of the pre-synaptic toxins and the venom concentration used being insufficient for eliciting such activity.

An important limitation of any in vitro experiment on antivenom efficacy is that it cannot be directly translated to the clinical effectiveness of antivenom. In these studies the antivenom was added to the organ bath 20 min prior to the venom, similar to pre-incubation studies. This is the ideal situation for testing antivenom and may not reflect the reality in treating patients with antivenom.

## 4. Conclusions

Using an in vitro nerve muscle preparation we have shown that the neurotoxicity of several clinically important Asian and Australian snake venoms is effectively cross-neutralised by a range of different antivenoms. There was a consistent pattern of cross-neutralisation of pre- and post-synaptic neurotoxicity suggesting that monovalent antivenoms raised against venoms with post-synaptic activity can cross-neutralise post-synaptic activity of other snake venoms and polyvalent antivenoms raised against both pre- and post-synaptic venoms can cross-neutralise both pre-and post-synaptic activity of different venoms. While acknowledging the limitation of adding antivenom prior to venom in an in vitro preparation, our study suggests that region-specific or universal neuro polyvalent antivenoms could be developed by raising antibodies against several carefully selected representative pre- and post-synaptic neurotoxins from snake venoms.

## 5. Materials and Methods

### 5.1. Venoms

The following snake venoms were used for the study: *Bungarus caeruleus* venom from Sri Lanka, *Bungarus fasciatus* venom from Thailand (Queen Saovabha Memorial Institute, Bangkok), *Ophiophagus hannah* venom from Indonesia (Venom Supplies, Tanunda, South Australia), *Naja kaouthia* (Venom Supplies, Tanunda, South Australia), and *Pseudonaja textillis*, *Oxyuranus scutellatus*, *Notechis scutatus*, *Acanthophis antarcticus* and *Pseudechis porphyriacus* (Venom Supplies, Tanunda, South Australia). Venom was dissolved in MilliQ water and stored at −20 °C until required. Protein quantification of the venom, fractions and toxins was carried out using a BCA Protein Assay Kit (Thermo Fisher Scientific, Rockford, IL, USA), as per the manufacturer’s instructions.

### 5.2. Neurotoxins

Using a single step reverse-phase high performance liquid chromatography (RP-HPLC) (LC-10ATVP pump and SPD-10AVP detector, Shimadzu, Kyoto, Japan, the short chain α-neurotoxins, α-scutoxin and α-elapitoxin-Ppr1 were isolated from *O. scutellatus* and *Pseudechis porphyriacus* venoms, respectively, as previously described in our laboratory [38,39]. Briefly, venoms were dissolved in solvent A (0.1% trifluoroacetic acid [TFA], Auspep, Tullamarine, VIC, Australia) and 2 mg of total protein was injected into a Phenomenex Jupiter semi-preparative C18 column (250 mm × 10 mm; 5 μm; 300 A˚, Phenomenex, Lane Cove, NSW, Australia), equilibrated with solvent A. Fractions were eluted using the following gradient of solvent B (90% acetronitrile [ACN, Merck KGaA, Darmstadt, Germany] in 0.1% TFA): 0%–20% over 5 min (4% gradient), 20%–60% over 5–45 min (1% gradient), and 60%–80% for 45–50 min (4% gradient) at a flow rate of 2.0 mL/min, monitoring at 214 nm. The long-chain α-neurotoxin, α-elapitoxin-Nk2a, was isolated and purified from *Naja kaouthia* venom using RP-HPLC following the same methodology described above. α-Bungarotoxin was purchased from Invitrogen (OR, USA; Batch no: 1601). The β-neurotoxin, taipoxin, was isolated and purified using size-exclusion chromatography, following the method described in Barber et al. [11]. Briefly, *O. scutellatus* venom (1 mg) was run through a Superdex G-75 column (13 μm; 10 mm × 300 mm; GE Healthcare, Uppsala, Sweden) equilibrated with ammonium acetate buffer (0.1 M, pH 6.8). The sample was run at a flow rate of 0.5 mL/min and was monitored at 280 nm.

The identity and the purity of the isolated toxins were further verified using matrix-assisted laser desorption/ionization (MALDI-TOF) mass spectrometry(4700 Proteomics Analyser MALDI TOF/TOF, AB Sciex, Foster City, CA, USA) as described previously [40,41]. HPLC and MALDI-TOF chromatograms are included as Supplementary Figure S1.

### 5.3. Antivenoms

Thai cobra (*Naja kaouthia*) monovalent antivenom (TCAV; Thai Red Cross Society, Bangkok, Thailand; Batch No: NK00111, date of expiry: 04.01.2016), death adder antivenom (DAAV; CSL, Parkville, VIC, Australia; Batch No: 07301, date of expiry: 09.2006), Indian polyvalent antivenom (IPAV; raised against *Bungarus caeruleus*, *Naja naja*, *Daboia russelii* and *Echis carinatus*; VINS Bioproducts, Andra Pradesh, India; Batch No: 01AS14001; date of expiry: 12.2017), Australian polyvalent antivenom (APAV; raised against *Psudechis australis*, *Notechis scutatus*, *Pseudonaja textillis*, *Acanthophis antarcticus* and *Oxyuranus scutellatus*; CSL, Parkville, VIC, Australia; Batch No: 18501, date of expiry: 02.2016) and Thai neuro polyvalent antivenom (TNPAV; raised against *Bungarus candidus*, *B. fasciatus*, *O. hannah* and *N. kaouthia*; Thai Red Cross Society, Bangkok, Thailand; Batch No: NP00113, date of expiry: 27.02.2018) were used for this study. According to the manufacturers of IPAV, TCAV and TNPAV, each antivenom vial should be dissolved in 10 mL of sterile water. The neutralization potential of the antivenom as stated on the labels are as follows: 1 mL IPAV for 0.45 mg *B. caeruleus* venom, 0.6 mg Indian cobra venom, 0.6 mg Russell’s viper venom, 0.45 mg Saw-scaled viper venom; 1 mL TCAV for 0.6 mg Thai cobra (*N. kaouthia*) venom; 1 mL TNPAV for 0.8 mg *O. hannah* venom, 0.6 mg *N. kaouthia* venom, 0.4 mg Malayan krait venom, 0.6 mg *B. fasciatus* venom. For Australian antivenoms which come in liquid form, 1 vial of APAV (34.4 mL) contains 1000 units of *P. textillis*, 6000 units of *A. antarcticus*, 18,000 units of *Pseudechis australis*, 12,000 units of *O. scutellatus* and 3000 units of *N. scutatus* antivenoms. One vial of DAAV (31.72 mL) contains 6000 units of *A. antarcticus* antivenom (1 unit neutralizes 1 µg venom).

### 5.4. Chick Biventer Cervicis Nerve-Muscle Preparation

Male chickens (aged 4–10 days) were humanely killed by exsanguination following CO_2_ inhalation. Biventer cervicis nerve-muscle preparations were dissected and then mounted on wire tissue holders under 1 g resting tension in 5 mL organ baths. Tissues were maintained at 34 °C, bubbled with 95% O_2_ and 5% CO_2_, in physiological salt solution of the following composition (mM); 118.4 NaCl, 4.7 KCl, 1.2 MgSO_4_, 1.2 KH_2_PO_4_, 2.5 CaCl_2_, 25 NaHCO_3_ and 11.1 glucose. Indirect twitches were evoked by stimulating the motor nerve (rate: 0.1 Hz; pulse duration: 0.2 ms) at supramaximal voltage (7–15 V), using a Grass S88 stimulator (Grass Instruments, Quincy, MA, USA). Selective stimulation of the nerve was confirmed by the abolishment of twitches with d-tubocurarine (10 µM, Sigma-Aldrich, St. Louis, MO, USA). Tissues were then repeatedly washed with physiological salt solution to restore twitch response to nerve stimulation. Contractile responses of the tissues to exogenous acetylcholine (ACh; Sigma-Aldrich, St. Louis, MO, USA; 1 mM for 30 s), carbachol (CCh; Sigma-Aldrich, St. Louis, MO, USA; 20 µM for 60 s) and KCl (40 mM for 30 s) were obtained in the absence of nerve stimulation. The preparations were then stimulated for 30 min, before the addition of antivenom. After the tissues were equilibrated with antivenom for 20 min, the venom or toxin was added. For each venom and toxin, observations were made for all antivenom experiments for at least the time at which the venom or toxin causes full twitch inhibition. At the conclusion of the experiment, ACh, CCh and KCl were re-added as above.

Initial experiments were carried out to select individual venom concentrations that fully abolish indirect twitches consistently, and based on those, all venoms, apart from *B. fasciatus* and *P. textillis* venoms were tested at 5 µg/mL. *B. fasciatus* venom was tested at 7.5 µg/mL and *P. textillis* venom was tested at both 1 µg/mL and 5 µg/mL. All toxins were tested at 100 nM. Each vial of TCAV and TNPAV antivenom was reconstituted with 10 mL of sterile water as instructed by the manufacturer. One vial of IPAV was reconstituted with 2 mL of sterile water, which is 5 times more concentrated than the manufacturer’s instructions. In order to achieve a sufficiently high concentration of antivenom for the venom, all antivenoms were tested at 40 µL/mL bath concentrations except IPAV which was tested at 15 µL/mL bath concentrations. Because the addition of large amounts of antivenom can alter the osmolarity of the physiological salt solution, antivenom control experiments (i.e., antivenom only) were performed to ensure the antivenom was not affecting the tissue viability.

### 5.5. Data Analysis and Statistics

Indirect twitch responses and responses to exogenous agonists (ACh, CCh and KCl) were measured via a Grass FTO3 force displacement transducer (Grass Instruments, Quincey, MA, USA) and recorded on a PowerLab system (ADInstruments Pty Ltd., Bella Vista, NSW, Australia). The t_90_ values (i.e., time taken for 90% inhibition of the maximum twitch response to occur) were determined for each of the venoms and the five toxins. All twitch and agonist responses were expressed as percentages of their pre-venom/toxin values. A one-way ANOVA was used to compare the responses to exogenous agonists following the administration of venom. All ANOVAs were followed by Bonferroni’s multiple comparison post-tests. Data are presented in the form of mean ± standard error of the mean (S.E.M.) of three to five experiments. All statistical analyses and presentation of data were generated using GraphPad Prism 6.07 software (GraphPad software Inc., La Jolla, CA, USA). For all statistical tests *p* < 0.05 was considered statistically significant.

### 5.6. Animal Ethics

All animal experiments used in this study were approved by the Monash University Animal Ethics Committee (Approval no: MARP/2014/097).

## Supplementary Materials

The following are available online at www.mdpi.com/2072-6651/8/10/302/s1, Figure S1: Chromatograms of HPLC and MALDI-TOF Mass spectrometry of the neurotoxins, Table S1: Comparison of the neurotoxicity of the venoms and neurotoxins in the absence of and presence of different antivenoms.

## References

[1] Kasturiratne A., Wickremasinghe A.R., De Silva N., Gunawardena N.K., De Silva N., Pathmeswaran A., Premaratna R., Savioli L., Lalloo D.G., De Silva H.J. (2008). The global burden of snakebite: A literature analysis and modelling based on regional estimates of envenoming and deaths. PLoS Med..

[2] Harrison R.A., Hargreaves A., Wagstaff S.C., Faragher B., Lalloo D.G. (2009). Snake envenoming: A disease of poverty. PLoS Negl. Trop. Dis..

[3] Gutiérrez J.M., Williams D., Fan H.W., Warrell D.A. (2010). Snakebite envenoming from a global perspective: Towards an integrated approach. Toxicon.

[4] Williams S.S., Wijesinghe C.A., Jayamanne S.F., Buckley N.A., Dawson A.H., Lalloo D.G., De Silva H.J. (2011). Delayed psychological morbidity associated with snakebite envenoming. PLoS Negl. Trop. Dis..

[5] Ranawaka U.K., Lalloo D.G., De Silva H.J. (2013). Neurotoxicity in snakebite-the limits of our knowledge. PLoS Negl. Trop. Dis..

[6] Hodgson W.C., Wickramaratna J.C. (2002). In-vitro neuromuscular activity of snake venoms. Clin. Exp. Pharmacol. Physiol..

[7] Harris J.B., Scott-Davey T. (2013). Secreted phospholipases A_2_ of snake venoms: Effects on the peripheral neuromuscular system with comments on the role of phospholipases A_2_ in disorders of the CNS and their uses in industry. Toxins.

[8] Prasarnpun S., Walsh J., Awad S.S., Harris J.B. (2005). Envenoming bites by kraits: The biological basis of treatment-resistant neuromuscular paralysis. Brain.

[9] Barber C.M., Isbister G.K., Hodgson W.C. (2013). Alpha neurotoxins. Toxicon.

[10] Nirthanan S., Gwee M.C.E. (2004). Three-finger-neurotoxins and the nicotinic acetylcholine receptor, forty years on. J. Pharmacol. Sci..

[11] Barber C.M., Isbister G.K., Hodgson W.C. (2012). Solving the “Brown snake paradox”: In vitro characterisation of Australasian snake presynaptic neurotoxin activity. Toxicol. Lett..

[12] Dixon R.W., Harris J.B. (1999). Nerve terminal damage by β-bungarotoxin. Am. J. Pathol..

[13] O’Leary M., Schneider J.J., Krishnan B.P., Lavis C., McKendry A., Ong L.K., Isbister G.K. (2007). Cross-neutralisation of Australian brown and tiger snake venoms with commercial antivenoms: Cross-reactivity or antivenom mixtures?. Toxicon.

[14] Tan C.H., Leong P.K., Fung S.Y., Sim S.M., Ponnudurai G., Ariaratnam C., Khomvilai S., Sitprija V., Tan N.H. (2011). Cross neutralization of *Hypnale hypnale* (hump-nosed pit viper) venom by polyvalent and monovalent Malayan pit viper antivenoms in vitro and in a rodent model. Acta Trop..

[15] Leong P.K., Tan N.H., Fung S.Y., Sim S.M. (2012). Cross neutralisation of Southeast Asian cobra and krait venoms by Indian polyvalent antivenoms. Trans. R. Soc. Trop. Med. Hyg..

[16] Leong P.K., Fung S.Y., Tan C.H., Sim S.M., Tan N.H. (2015). Immunological cross-reactivity and neutralization of the principal toxins of *Naja sumatrana* and related cobra venoms by a Thai polyvalent antivenom (Neuro Polyvalent Snake Antivenom). Acta Trop..

[17] Isbister G.K., Maduwage K., Page C.B. (2014). Antivenom cross neutralisation in a suspected Asian pit viper envenoming causing severe coagulopathy. Toxicon.

[18] Kornhauser R., Isbister G.K., O’Leary M.A., Mirtschin P., Dunstan N., Hodgson W.C. (2013). Cross-neutralisation of the neurotoxic effects of Egyptian cobra venom with commercial tiger snake antivenom. Basic Clin. Pharmacol. Toxicol..

[19] Leong P.K., Sim S.M., Fung S.Y., Sumana K., Sitprija V., Tan N.H. (2012). Cross neutralization of Afro-Asian cobra and Asian krait venoms by a Thai polyvalent snake antivenom (neuro polyvalent snake antivenom). PLoS Negl. Trop. Dis..

[20] Petras D., Sanz L., Segura Á., Herrera M., Villalta M., Solano D., Vargas M., León G., Warrell D., Theakston R.D.G. (2011). Snake venomics of African spitting cobras: Toxin composition and assessment of congeneric cross-reactivity of the Pan-African EchiTAb-Plus-ICP antivenom by antivenomics and neutralization approaches. J. Proteome Res..

[21] Maduwage K., Silva A., O’Leary M.A., Hodgson W.C., Isbister G.K. (2016). Efficacy of Indian polyvalent snake antivenoms against Sri Lankan snake venoms: Lethality studies or clinically focussed in vitro studies. Sci. Rep..

[22] Silva A., Maduwage K., Sedgwick M., Pilapitiya S., Weerawansa P., Dahanayaka N.J., Buckley N.A., Johnston C., Siribaddana S., Isbister G.K. (2016). Neuromuscular effects of common krait (*Bungarus caeruleus*) envenoming in Sri Lanka. PLoS Negl. Trop. Dis..

[23] Johnston C.I., O’Leary M., Brown S.G., Currie B.J., Halkidis L., Whitaker R., Close B., Isbister G.K. (2012). Death adder envenoming causes neurotoxicity not reversed by antivenom-Australian Snakebite Project (ASP-16). PLoS Negl. Trop. Dis..

[24] Silva A., Maduwage K., Sedgwick M., Pilapitiya S., Weerawansa P., Dahanayake N., Buckley N.A., Siribaddana S., Isbister G.K. (2016). Neurotoxicity in Russell’s viper (*Daboia russelii*) envenoming in Sri Lanka: A clinical and neurophysiological study. Clin. Toxicol..

[25] Watt G., Meade B.D., Theakston R.D.G., Padre L.P., Tuazonl M.L., Calubaquib C., Santiago E., Ranoa C.P., Naval U.S., No U. (1989). Comparison of Tensilon and antivenom for the treatment of cobra bite paralysis. Trans. R. Soc. Trop. Med. Hyg..

[26] World Health Organisation (2010). WHO Guidelines for the Production Control and Regulation of Snake Antivenom Immunoglobulins.

[27] Gutiérrez J.M., León G., Burnouf T. (2011). Antivenoms for the treatment of snakebite envenomings: The road ahead. Biologicals.

[28] Cardoso R., Homsi-Brandeburgo M.I., Rodrigues V.M., Santos W.B., Souza G.L.R., Prudencio C.R., Siquieroli A.C.S., Goulart L.R. (2009). Peptide mimicking antigenic and immunogenic epitope of neuwiedase from *Bothrops neuwiedi* snake venom. Toxicon.

[29] Fry B.G., Wüster W., Kini R.M., Brusic V., Khan A., Venkataraman D., Rooney A.P. (2003). Molecular evolution and phylogeny of elapid snake venom three-finger toxins. J. Mol. Evol..

[30] Antil S., Servent D., Menez A. (1999). Variability among the sites by which curaremimetic toxins bind to torpedo acetylcholine receptor, as revealed by identification of the functional residues of alpha-Elapitoxin-Nk2a. J. Biol. Chem..

[31] O’Leary M.A., Isbister G.K. (2009). Commercial monovalent antivenoms in Australia are polyvalent. Toxicon.

[32] Kini R.M. (2003). Excitement ahead: Structure, function and mechanism of snake venom phospholipase A_2_ enzymes. Toxicon.

[33] Barfaraz A., Harvey A.L. (1994). The use of the chick biventer cervicis preparation to assess the protective activity of six international reference antivenoms on the neuromuscular effects of snake venoms in vitro. Toxicon.

[34] Sharma S., Karthikeyan S., Betzel C., Singh T.P. (1999). Isolation, purification, crystallization and preliminary X-ray analysis of β_1_ -bungarotoxin from *Bungarus caeruleus* (Indian common krait). Acta Crystallogr. Sect. D Biol. Crystallogr..

[35] Rusmili M.R.A., Yee T.T., Mustafa M.R., Hodgson W.C., Othman I. (2014). Proteomic characterization and comparison of Malaysian *Bungarus candidus* and *Bungarus fasciatus* venoms. J. Proteomics.

[36] Halpert J., Eaker D. (1975). Amino acid sequence of a presynaptic neurotoxin from the venom of *Notechis scutatus*. J. Biol. Chem..

[37] Tan K.Y., Tan C.H., Fung S.Y., Tan N.H. (2015). Venomics, lethality and neutralization of *Naja kaouthia* (monocled cobra) venoms from three different geographical regions of Southeast Asia. J. Proteomics.

[38] Kornhauser R., Hart A.J., Reeve S., Smith A.I., Fry B.G., Hodgson W.C. (2010). Variations in the pharmacological profile of post-synaptic neurotoxins isolated from the venoms of the Papuan (*Oxyuranus scutellatus canni*) and coastal (*Oxyuranus scutellatus scutellatus*) taipans. Neurotoxicology.

[39] Hart A.J., Isbister G.K., O’Donnell P., Williamson N.A., Hodgson W.C. (2013). Species differences in the neuromuscular activity of post-synaptic neurotoxins from two Australian black snakes (*Pseudechis porphyriacus* and *Pseudechis colletti*). Toxicol. Lett..

[40] Silva A., Kuruppu S., Othman I., Goode R.J.A., Hodgson W.C., Isbister G.K. (2016). Neurotoxicity in Sri Lankan Russell’s viper (*Daboia russelii*) envenoming is primarily due to U1-viperitoxin-Dr1a, a pre-synaptic neurotoxin. Neurotox. Res..

[41] Chaisakul J., Konstantakopoulos N., Smith A., Hodgson W. (2010). Isolation and characterisation of P-EPTX-Ap1a and P-EPTX-Ar1a: Pre-synaptic neurotoxins from the venom of the northern (*Acanthophis praelongus*) and Irian Jayan (*Acanthophis rugosus*) death adders. Biochem. Pharmacol..

