# Supplementary material for: Cross-Neutralisation of In Vitro Neurotoxicity of Asian and Australian Snake Neurotoxins and Venoms by Different Antivenoms
Source: Toxins (Basel). 2016 Oct 18;8(10):302. doi: 10.3390/toxins8100302 (PMC5086662; doi:10.3390/toxins8100302)
Supplement: Supplementary file 1 [file toxins-08-00302-s001.pdf]

Supplementary Materials: In-Vitro Cross-Neutralisation of the Neurotoxicity of Asian and Australian Snake Neurotoxins and Venoms by Different Antivenoms

Anjana Silva, Wayne C. Hodgson and Geoffrey K. Isbister

Table S1. Comparison of the neurotoxicity of the venoms and neurotoxins in the absence of and presence of different antivenoms.

Treatment	*Bungarus caeruleus* (5 µg/mL)		*B. fasciatus* (7.5 µg/mL)		*Naja naja* (5 µg/mL)		*Ophiophagus hannah* (5 µg/mL)		*Notechis scutatus* (5 µg/mL)		*Oxyuranus scutellatus* (5 µg/mL)		*Acanthophis antarcticus* (5 µg/mL)		*Pseudonaja textillis* (5 µg/mL)		α-Cobratoxin (100 nM)		α-Bungarotoxin (100 nM)		α-Elapitoxin-Ppr1 (100 nM)		α-Scutoxin (100 nM)		Notexin (100 nM)	
	t_{90}	SD	t_{90}	SD	t_{90}	SD	t_{90}	SD	t_{90}	SD	t_{90}	SD	t_{90}	SD	t_{90}	SD	t_{90}	SD	t_{90}	SD	t_{90}	SD	t_{90}	SD	t_{90}	SD
**No Antivenom**	35.0	3.3	69.0	6.7	18.0	2.2	58.0	5.6	30.0	1.3	128.0	17.2	22.5	1.8	15.0	2.1	30.3	3.4	11.1	2.0	31.5	3.9	40.5	4.3	108.3	14.6
**TCAV**	44.0	1.4	FP		FP		FP		PP		136.6	7.2	FP		22.0	4.5	FP		FP		FP		PP		N/T	
**TNPAV**	FP		FP		FP		FP		FP		FP		FP		23.0	5.5	N/T		N/T		N/T		N/T		FP	
**IPAV**	FP		FP		PP		FP		FP		PP		PP *		23.0	5.5	N/T		N/T		N/T		N/T		125.0	2.5
**APAV**	FP		FP		FP		FP		FP		FP		FP		FP		FP		FP		FP		PP		FP	
**DAAV**	FP		FP		FP		FP		FP		FP		FP		FP		N/T		N/T		N/T		N/T		N/T	

Note: t_{90} is provided in instances where >90% of twitch height was inhibited; SD: Standard deviation. TCAV: Thai cobra antivenom; TNPAV: Thai neuro polyvalent antivenom; IPAV: Indian Polyvalent antivenom; APAV: Australian polyvalent antivenom; DAAV: death adder antivenom. FP: Full prevention of twitch inhibition; PP: Partial prevention of twitch inhibition; N/T: Not tested; * >85% twitch inhibition.

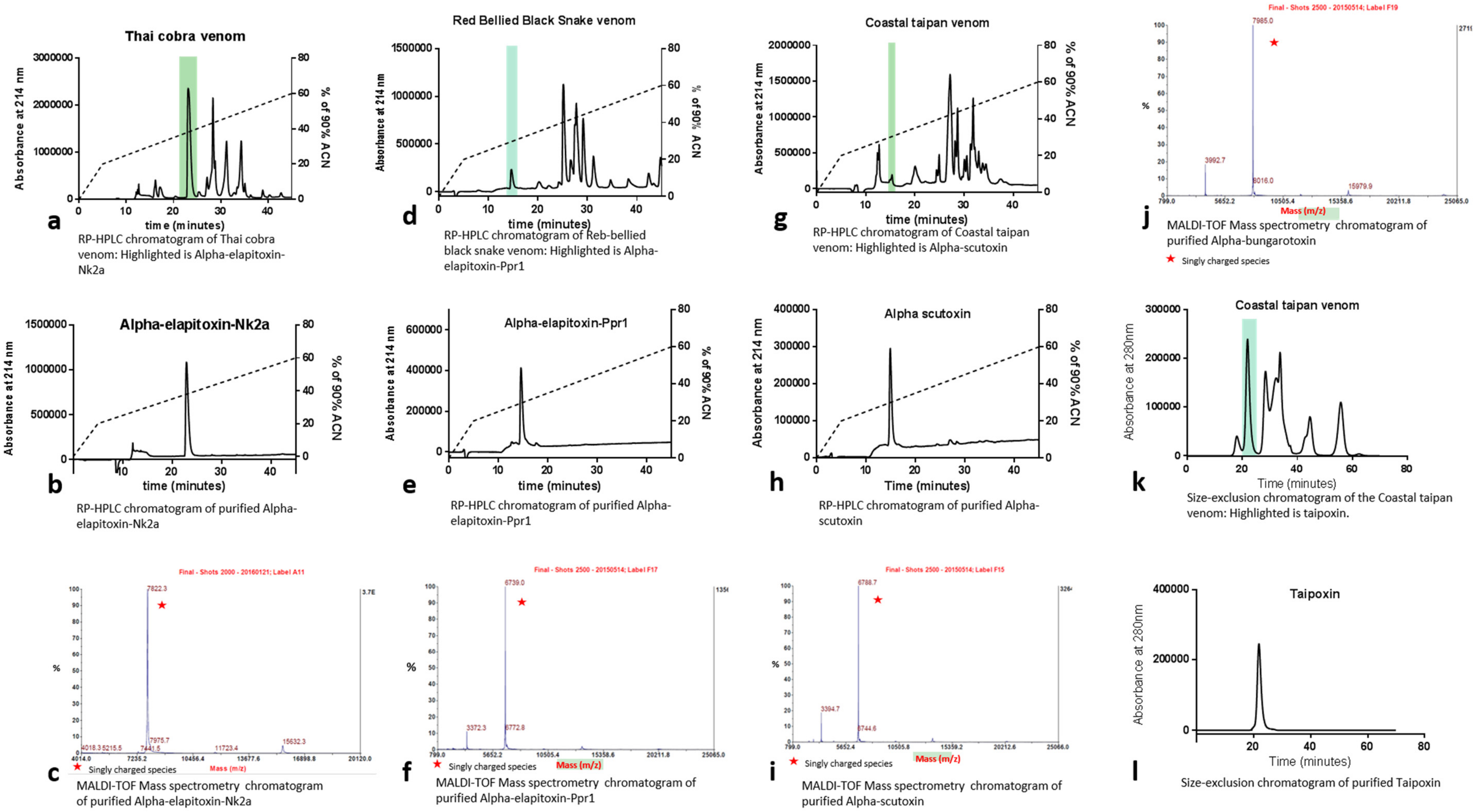

Figure S1. Chromatograms of HPLC and MALDI-TOF Mass spectrometry of the neurotoxins.
